# Assessing the effects of gold mining on environment: A case study of Shekiso district, Guji zone, Ethiopia

**DOI:** 10.1016/j.heliyon.2022.e11882

**Published:** 2022-11-26

**Authors:** Birhanu Bekele Mencho

**Affiliations:** Department of Geography and Environmental Studies, Injibara University, Awi, Po Box 40, Ethiopia

**Keywords:** Environmental sustainability, Destruction, Guji zone, Shekiso district, Study area

## Abstract

Environmental sustainability has become a serious problem in the world. Similarly, on environmental of the mining in the Shekiso district has become a severe issue at present. Such events have fueled an often contentious debate about how to identify areas that should be declared off-limits to mining because of too-rapid social and environmental sensitivity. Therefore, this study aims to assess the effects of the gold mining on the environment at a selected kebele in the case of Shekiso District, Guji Zone, Ethiopia. The primary data used for this study was obtained from 283 randomly selected sample in the study area. This data was collected using a structured interview, focus group discussions, observation, and key informant interviews, then analyzed using descriptive statistics. Besides, a semi-structured interview was used to collect data from the mining and energy office in Shekiso District. The key informant was selected specifically to infer the effects of gold mining on the environment in the study area. The survey results indicate that mining serves as a key source of income (53%), a source of raw materials (30%), and employment (17%). On the other hand, gold mining is a root cause of environmental problems such as water shortages (8.8%), dehydration of the brook (10.6%), soil erosion (20.8%), damage to the street (17.6%), and destruction of the ecosystem (7.0%). Besides that, about 8.8%, 8.8%, and 6.3% of households stated that mining operations cause deforestation, air pollution, and destruction of aquatic life, respectively. Generally, due to a lack of environmental awareness programs through education in many gold mining communities, safeguarding sustainable use of the natural environment p*oses several challenges in study area*. As a result, local governments should raise awareness, facilitate registration, and address rules and enforcement in an effort to enhance ecologically friendly mining.

## 1 Introduction

In different parts of the world, the gold mining activity plays a great role in enhancing livelihoods (Ontoyin and Agyemang, 2014; Djangmah, 2016). It is estimated that thirteen million households were directly involved in mining operations around the world. Macro-scale mining operations provide sources of employment, income, and foreign currency (Wale et al., 2021; Mkodzongi et al., 2019; Mkodzongi et al., 2019, 2019). This is due increasing consumption of the gold, iron, as well as other mining raw materials throughout the globe (Rukmana et al., 2020, and Krutilla, 2021). Even though, mining simulates vital economic growth and development, unattainable mining operations can harm the environment.

Moreover, if mining neglects the environment, it harms the environment and leads to environmental demolition (Novianantya et al., 2017). It results land destruction, soil erosion, water pollution, ecosystem destruction, and etc. (Haddaway et al., 2019; Novianti et al., 2017). Also, it affects the health and well-being of society, individuals, and the public at large due to the foxiness of the environment created by unsustainable mining operations (Novianantya et al., 2017; Haddaway et al., 2019; Wale et al., 2021).

Generally, unsustainable mining has significant adverse impacts on society, the economy, and the environment directly (Tutu, 2014). As a result, it is better for the mining industry to develop strategies to mitigate harmful environmental effects (Barbosa et al., 2021; and Belayneh et al., 2021).

Ethiopia is rich in gold deposits, which are found in the north, south, and west parts of the country (Ismail et al., 2019). The government of Ethiopia has been producing gold since the late 1930s as a state-owned gold mine. Since 1997, the state-owned Guji zone gold mining has been privatized by the MIDROC PLC gold company. Beside that since the mid-1920s, the Guji zone has been exploited for its mineral resources without regard to environmental sustainability. Inline with these finding Asantewaa et al. (2019) suggest that, the Guji zone faces several environmental challenges, such as soil erosion, land degradation, and contamination of water due to a lack of environmental policies and responsibility related to mining activity. Yet, to a large extent, the accountability and authorization of extractive industries for the impact and consequences of environmental degradation have been very poor (Wale et al., 2021).

Consequently, these operations remain negatively squeezed on a daily basis (Pramono, 2014; Rai et al., 2014; Wale et al., 2021; Asantewaa et al., 2019). Although the magnitude of the environmental concerns caused by gold mining in *Shekiso* is not well documented, little study has gone into measure to remedy this issue. Studies on mining-related environmental concerns focused on ways to develop an optimum mining design to meet future needs and minimize ore losses. For example, Haile and Konka (2021) conducted research on Optimum Open Pit Design for *Kenticha Tantalite* Mine, Southern Ethiopia. Also, regassa, (2021) conducted research on the frontiers of extraction and contestation: dispossession, exclusion, and local resistance against *Laga-Dambi* Gold Mine, southern Ethiopia. This paper focused on how questions of entitlement at the local level, mining micro politics, and the national political order are entangled and produce different forms of contestation and negotiation. It reaches the conclusion that this embarrassment influences how mining corporations and their activities are governed. However, very little effort was considered to mitigate the environmental effects of gold mining, and yet, no attempts have been made to identify what might constitute vulnerable ecosystems. Unfortunately, there is a great deal of ambiguity in determining whether the potential environmental and social costs of mining are too high. Thus, by widening the subject's knowledge range, by investigating how local populations would accept the task and responsibility of sustaining degraded landscapes as de facto owners of natural resources within the mining environment, Therefore, researchers are motivated to study the effects of mining on the environment in the selected kebele in the Shekiso district. To that end, the study will provide a better understanding of mining communities' attitudes and perceptions toward the impact of small-scale gold activities on ecosystem services, as well as their willingness to participate in recovery programs aimed at improving ecosystem services in order to sustain rural livelihoods. As a result, the study aims to assess the effects of the gold mining on environment and to examine perception of local communities on environments as well as to investigate the contribution of the mining to the local households in study area.

## 2 Materials and methods

### 2.1 Description of the study area

**Figure 1:**
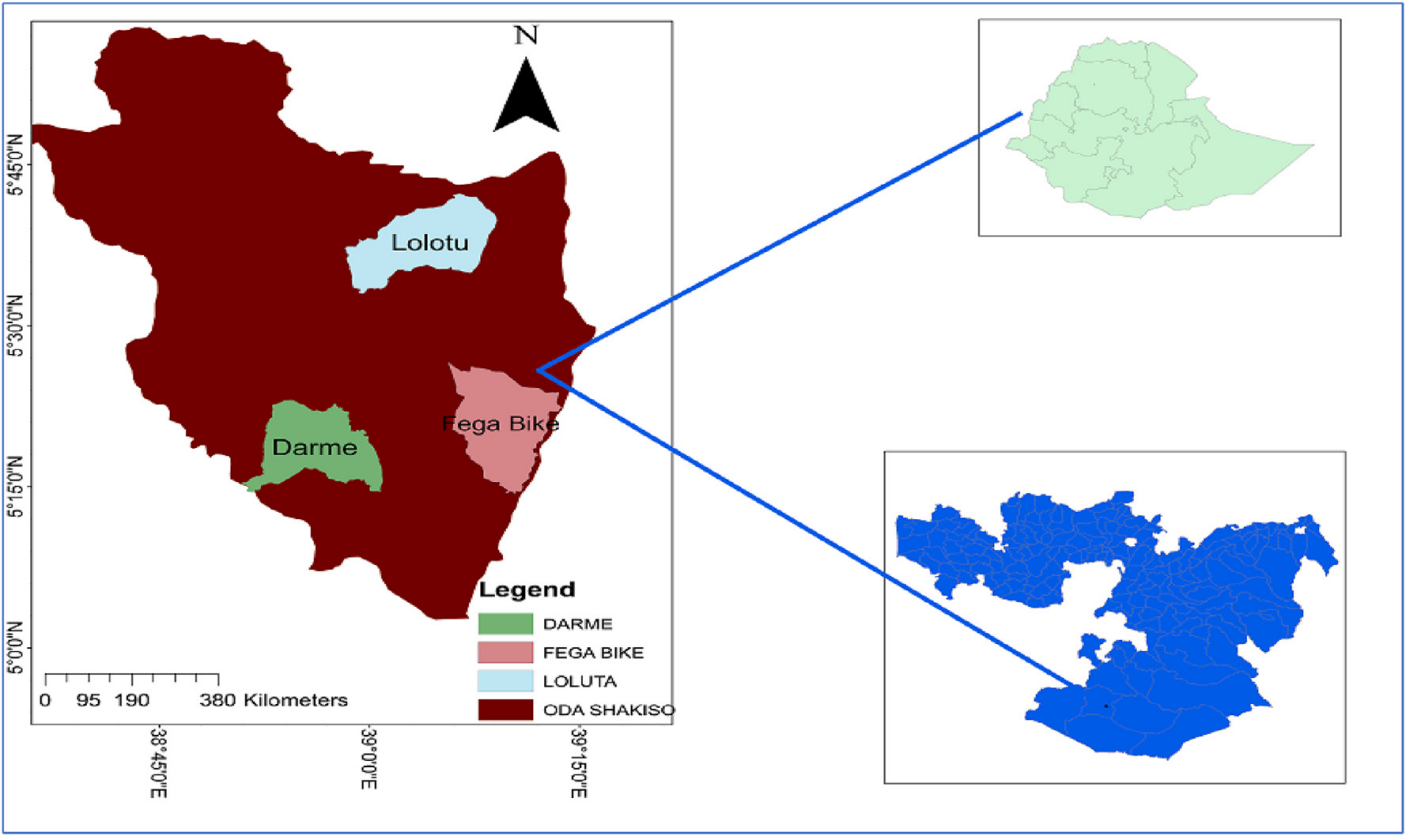
Map of the study area. Sources: Arc GIS 10.7.

*Shekiso* District is in the Oromyia Region, which is located at 5°15′0″–5°45′0″ N and 38°45′0″–39°30′ 0″ E, with an elevation of 1758 m above sea level (Figure 1). According to CSA (2017), the projected district's population is 259,641, specifically 134,523 males and 125,118 females. The climate of the district was characterized by an equatorial type with two rainfall characteristics, meaning from February to July is a rainy month, while September to December is a dry month (Ayele et al., 2021). The temperature of the district is high, with records showing significant seasonal and daily variations in all respects. The annual temperature ranges between 26 and 29 °C.

Major socio-economic activities in the study area include mixed farming, large-scale and small-scale gold mining. Most of the population (90%) is engaged in farming activity, which provides their main source of livelihood (Tarekegn and Terefe, 2019). Crops grown in the districts are mostly cash crops and subsistence. Subsistence farming, such as vegetable and cash crop cultivation, is practiced in Shikiso Districts. Cash crop production mainly involved cultivation of the palm crop, coffee, and Khat.

### 2.2 Sampling procedures and data collection

This study followed a multi-stage sampling procedure. Due to the large coverage of mining activity and gold resource availability, the first stage gold mining communities, such as *Guji* zone, were chosen at random among the *Borena* Zones. Second, the *Shekiso* district was purposefully chosen among the *Guji* Zone districts due to its extensive coverage of gold mining activity and environmental degradation. In the third stage, three *Kebele* administrations, namely *Drame, Fegabike*, and *Lolotu,* were selected using a purposeful sampling method due to the high extent of mining activity. Finally, at the third stage, the sampling was determined by considering different factors into account, such as time, research cost, accessibility and availability of transport, 283 sample households were included in the sample. The sample respondents was selected from the three kebele using a simple random sampling method by a proportional to size technique out of 941 total households residing within the study area. The choice of the sampled household was made using a simplified formula to derive sample size at a 95% confidence level with 0.03% error (Table 1).

$$n=\frac{Z^{2}.P.q.N}{e^{2}\left(N-1\right)+z^{2}\;P.q}$$

$$n=\frac{1.96^{2}x0.05x0.95x\;941}{0.03^{2}\left(941-1\right)+1.96^{2}\;0.05x0.95}=283$$

where N = the total households = 941, n = size of sample, e = acceptable error (0.03), z = standard variant a given confidence level (1.96) 95% confidence, p = (0.05) population proportion, q = 1 − p (1–0.05 = 0.95).

**Table 1:** Stratified sampling.

Kebele	Total household per kebele adminstraion	Number of samples
Lolouta	340	102
Fegabike	325	98
Darme	276	83
Total households	941	**283**

Source: Shekiso District Agriculture and Finance Offices (2021**)**.

The based on a formula used by sample of each *kebele* was determined (Berafe, 2017):

$$n1=\frac{N1}{N}\left(n\right)$$

were; n1 = the size of sample each kebele adminstration, N1 = the size of population each kebele adminstration, N = the size of the total population, n = the size of total sample required, n_1_ = 325/ 941 × 283 = 98 (*Fegabike kebele*), n_2_ = 340/ 941 × 283 = 102 (*Lolouta kebele*), n_3_ = 276/941 × 283 = 83 (*Darme Kebele*) = the total sample size of the household was 283 (Table 1).

The required data for these studies was collected from secondary and primary data sources. The primary data source was questionnaires, interviews, observation, document analysis and focus group discussion. This focused on the effects of the gold mining on environment and to examine perception of local communities on environments as well as to investigate the contribution of the mining to local households in study area. A secondary data source includes books, published literature related to effects of mining operation to environment. A simple random sampling method was used to select respondents from local mining societies. The data was collected in 2021 through face-to-face interviews using questionnaires from households. Were as this research was employed with mixed approaches to collect data from representative households. It is the most important tool to cover a large sample with the least cost (Asantewaa et al., 2019; Taufiq et al., 2018). The researcher used analogous questionnaires for all respondents to each selected *kebele* by using semi-structured and structured interviews. Moreover, the researcher used thirty household for initial survey from sampled *kebele* to check the rationality of the prepared questions for data collection. Then, after necessary modification following the reaction from the initial survey, the questionnaire was organized in English language. Therefore, the data collector was employed basis on their ability in the communicating *Oromiffa* language, educational background, and previous involvements in similar research work. Concerning data collection methods, training, supervision, and direction were given by the researcher on basic steps to be followed in the process of accompanying them during data collection.

Focus group discussions are most effective data collection tools (Teshome, 2018). Thus the researcher use, seven individuals were purposively selected by researcher and included in discussion. It is important means of stimulating views and opinions on particular issue. In these cases the researcher was acts as facilitator. Participants described their own opinions and knowledge or skills, but they also listened from others and reflect what is said. The respondents for focus group discussions were farmers, and gold miners. The discussion focused on the research issues in relation to the effects of gold mining on environments and its benefits to household in study area. Moreover, this method was carried out among groups of selected model farmers in the study area.

Key informant interviews were held with the local administrations, energy and mining experts, and natural resources management experts to gain a deeper understanding of the effects of mining in the study area. One key informant from natural resources management experts, one from *Kebele* administration, and two from energy and mining experts were selected purposively as they were considered representative of the population based on social class, age, gender, and local knowledge. The key informants were selected At *Kebele* level, four of the key informants were purposefully selected from each *Kebele* to gain an overview of the general information about the effects of mining on the environment and to examine perception of local communities on environments as well as the investigate the contribution of the mining to local households in study area. Key informants who stay more in districts were selected to be interviewed due to their better acquaintance or good knowledge of the local effects of mining on the environment. Therefore, the researcher used structured and semi-structured interviews to collect valuable information from respondents to support and fill the gaps in the study.

Observation is one of the scientific instruments for researchers to collect data. Under this, the information was sought by the investigator without asking others. Thus, observation is the most important tool for data collection relevant to a phenomenon or situation. It is the most important tool to collect qualitative data and record events, behaviors, and objects in a natural setting (Haddaway et al., 2019). For this study, the researcher recorded what he saw at the mining site about the environmental effects of mining. Observation helps the researcher explain the existing mining activity and compare the reported information with the actual occurrence in the study area.

### 2.3 Research design

In situation where there is no well-developed system for gathering and handling production data, it is difficult to get reliable time-series production data of individual small-scale farmers through interview. As a result, the study was undertaken taking cross-sectional data for crops. Thus these studies used a cross-sectional survey research design because it is best to study the prevalence of the mining community's situation across the population in the study area. Cross-sectional research design is descriptive and very important to look at how things are at present without any sense of history or trendsetting, and it reduces money and time wastage (Tutu, 2014).

### 2.4 Method of the data analysis

Data obtained from the household survey was analyzed using the SPSS version 20 computer software program. Before analysis, gathered questionnaires were coded, entered, and arranged. After coding was completed, all valid questionnaires were input into a coherent format in the SPSS database. Then, quantitative data was clarified by using descriptive statistics like frequency and percentages. The qualitative data was analyzed both descriptively in a kind of narration and qualitatively in a combined method relying on the obtained data for explaining, refuting, enriching, and confirming data. The analysis is supplemented with visual photographs recorded during field observation to characterize the major effects of mining on vegetation, water, land, etc. This quantitative and qualitative data gathered from household surveys, field observation, key informant interviews, and focus discussion was used to describe the environmental effects of mininig on the environment and to state perception of local communities on environments as well as to the describe the contribution of the mining to local households in study area. Also, secondary data gathered from various agricultural documents was organized and analyzed to support the survey results.

The results were then presented in the form of tables, figures, discussion cross-tabulations, and photographs. The research participants included in this study were appropriately informed about the purpose of the research and their consent was secured before the commencement of the interviews. Concerning the right to privacy of the respondents, the study maintained the secrecy of the uniqueness of each participant. Regarding data or information, the valuable data or information about mining has been obtained from Shekiso's woreda and has informed consent with Shekiso's District health center, Shakisso District Administration, Mineral Administration and Licensing Directorate, Borena Zone Forest and Wildlife Protection Agency, and Ministry of Mines; Environmental and Community Development Directorate.

## 3 Results and Discussion

### 3.1 Demographic and personal characteristics of sampled household

According to survey result, about 67% of the households were males while the rest 33% of them are females (Table 2). Regarding to ages of the household, about 29.3% of the households was fall to age of 24–28 years, while 20.8% of the between were categorized to age between 29-33 year (Table 2). This shows most of the households were categorized under the age group of 19–23 years. Whereas 85% of the households were categorized literate and the remaining 15% of the households were illiterate. In addition to that, 36% of the households were engaged in farming activity, 35.3% work as labor workers, while reaming 28.6% of households were civil servants. Furthermore, occupation of household data in. clearly indicates that 31.4% of the households were married while 36.7% of households were divorced and single 31.8%.

**Table 2:** Characteristics of sampled households.

	Age of household	Frequency	Percent
Age household	19–23	100	35.3
	24–28	83	29.3
	29–33	59	20.8
	34 and above	41	14.4
Sex of household	Male	190	67
	Female	93	33
Occupation of household	Farmer	102	36
	Civil servant	81	28.6
	Labor worker	100	35.3
Education level of household	Illiterate	240	85
	Literate	43	15
Marital status of household	Married	89	31.4
	Single	90	31.8
	Divorced	104	36.7

Sources: Household survey, 2021

### 3.2 Environmental impacts of gold mining

As findings indicate in (Table 3) that overall impacts of the mining operations the dehydration of the brook (10.6%), soil erosion (20.8%), and damage of the street (17.6%) are major consequences of the mining operations in study area. Extracting gemstones and gold mining negatively affects the environment through destruction of the ecosystem, depletion of water quality, and loss of vegetation (Belayneh et al., 2021).

**Table 3:** Environmental impacts of the gold mining.

Response of the households	Frequency	Percent
Water shortage	25	8.8
Dehydration of the brook	30	10.6
Soil erosion	59	20.8
Damage of street	50	17.6
Destruction of ecosystem	20	7.0
Deforestation	25	8.8
Water contamination	31	10.9
Air effluence	25	8.8
Loss of the aquatic life	18	6.3
Total	100	100%

Sources: Household survey, 2021

#### 3.2.1 Gold mining's impact on water resources

It is evident from the soil test results that the arsenic levels in the rivers were extremely high, above the maximum concentration levels allowed by the World Health Organization (WHO) for drinking water. The free cyanide concentrations in the Drame and Fega Rivers, as well as the Lokotu and Dawa Rivers, were slightly higher than the WHO guideline values (Table 4). Likewise, lead concentrations for all the rivers were also above both world health organizations (WHO) and EPA standardized levels (WHO, 2019; Barnhart et al., 2021 and Parsoya, 2022, ​Regassa, 2021).

**Table 4:** Sample Test of Polluted Rivers in study area.

Soil Testing date	Sample of soil	PH	CN(F) (mg/l)	As (mg/l)
04/3/2021	Drame River	7.6	6.20	0.21
04/3/2021	Fega River	7.3	3.50	0.20
04/3/2021	Lokotu River	7.7	1.42	<0.04
04/3/2021	Dawa River	7.1	2.65	<0.02
WHO value	6.5–8.5	0.05 mg/l	0.2 mg/l	0.01 mg/l

**NB**: pH- Acidity, As-Arsenic CN(F)-Cyanide Free.

**Source**: Shakiso District Environmental Department, 2021.

**Figure 2:**
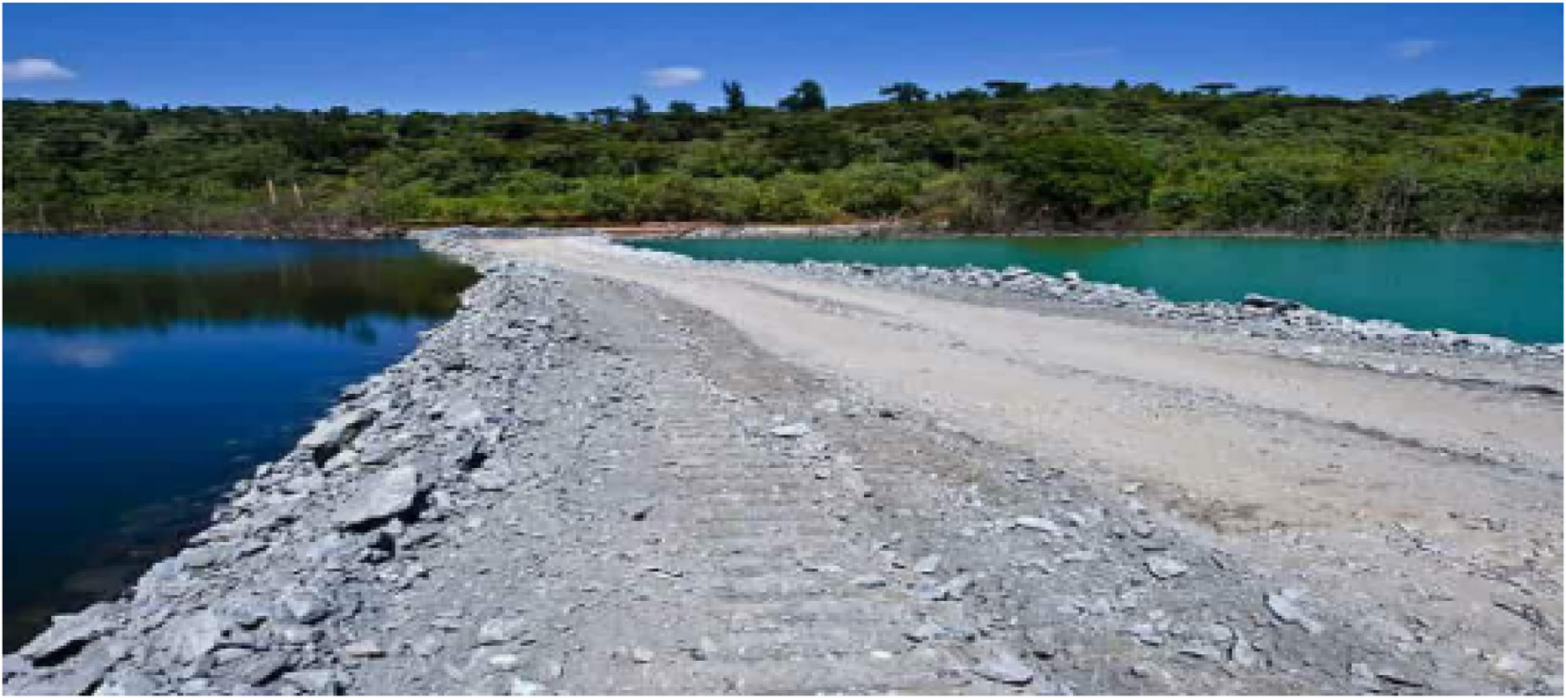
Impacts of mining on water quality. Sources: Filed observation, 2021.

The field observations show that the river serves as the chief source of drinking water for the households in the study area. However, it has been highly polluted by mining activities. The gold mining as well as minerals left unfilled, which are not suitable for any other uses, results in wide breeding surfaces for malaria-infected mosquitoes that have a significant negative impact on human health (Figure 2). Moreover, the key informant's interview shows that, major river heads have dried due to extensive gully and sheet erosion washing the sediments downstream of the river due to local mining operations. This finding was confirmed by the fact that areas known for mining operations are highly prone to soil erosion due to a lack of vegetation cover (Fitri, 2019; Macháček, 2020; Rukmana et al., 2020).

**Figure 3:**
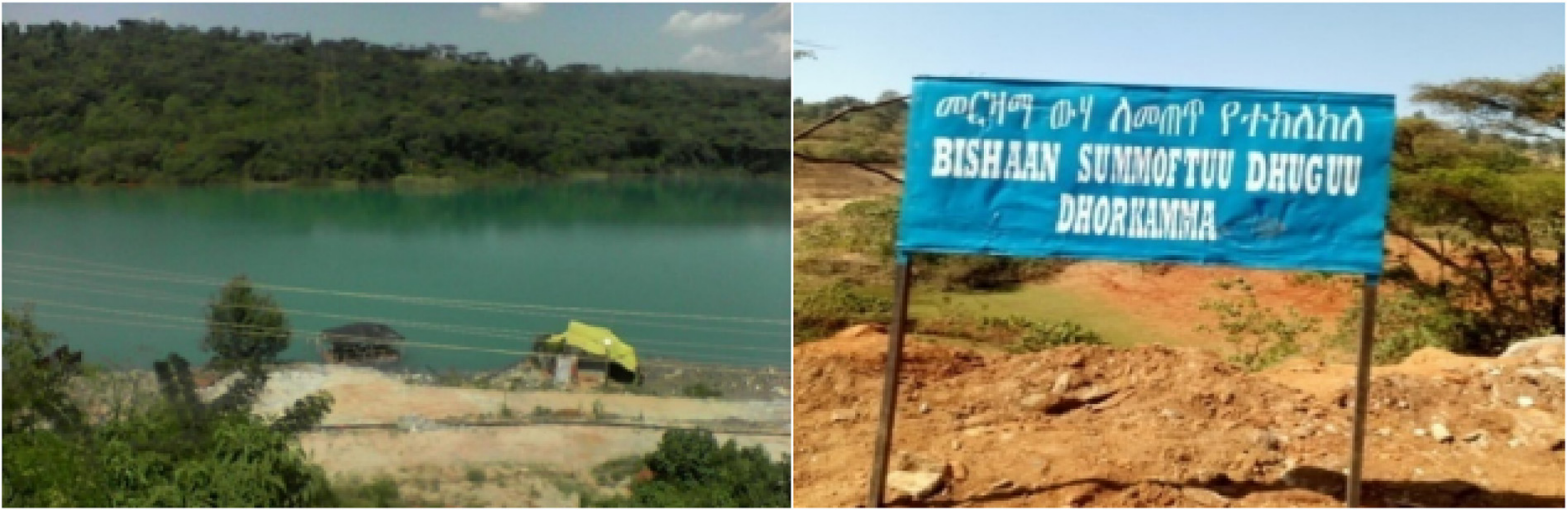
Acidic and polluted water. Souces: Cherinet, 2018.

In particular, the shallow mining operation has consequences that bear antagonistic environmental effects on water bodies such as streams and rivers through discharging solid suspended materials such as arsenic, mercury, and others (Figure 3). Because of the lack of vegetation, surface mining activity causes rock and mineral crystals to dissolve into water and be transported by running water, such as a river, resulting in water pollution. It is a more prevalent phenomenon in Ethiopia's highlands and poses a serious threat to subsurface and subsurface water bodies (Rai et al., 2014). Surface mining is very controversial due to its significant impact on terrain, flora, and water supplies (Sankararamakrishnan et al., 2005; Yang et al., 2013) Degradation, biodiversity loss, and pollution in various forms are some of the problems. If preventive steps are not undertaken, surface mining can have negative consequences for nearby groundwater and surface water (Ochieng et al., 2010). As a result, unusually high concentrations of some chemical elements, particularly arsenic and sulphuric acid, can exist over a broad surface and subsurface area.

As we understood from the above picture, the left-side dark cyanide-contaminated water was released by the side of the unpolluted water level (Figure 3). This indicates that the surface and underground water bodies in the study area are plagued by toxic chemical disposal and that their adjacent areas are polluted (Figure 3). The Shekiso district residents was counseled to avoid useing of river for daily activities likes for safety and health reasons.

### 3.3 Impacts of gold mining on vegetation and landscape

**Figure 4:**
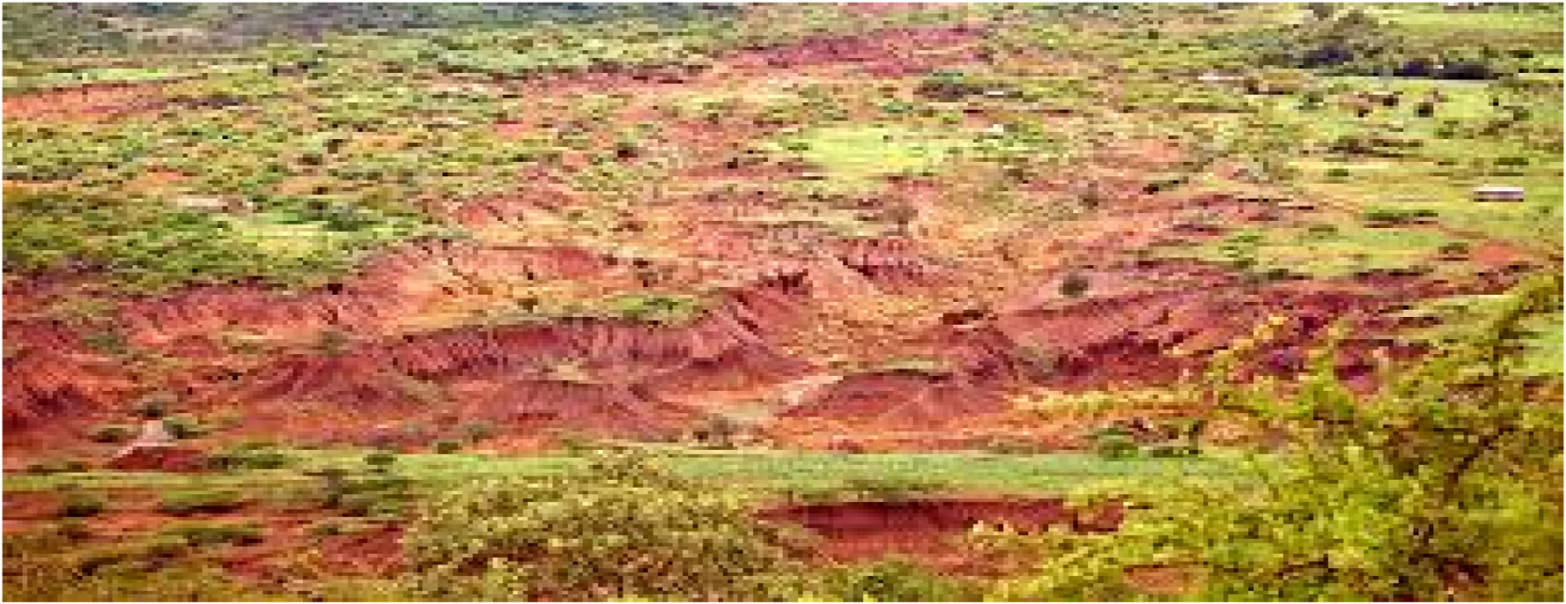
Impacts of gold mining on vegetation and landscape.

Long-term small and large-scale gold mining operations have been responsible for the deletion of massive amounts of surface luxuriant vegetation, and their mass destruction affect the biodiversity in a natural setting. Thus loss of ecological services triggered by disorders in terrestrial ecosystems that cannot recuperate again (Tsurukawa et al., 2011). It resulted in a broad section of the surface areas remaining as bare land in the study area, as shown in Figure 4.

**Figure 5:**
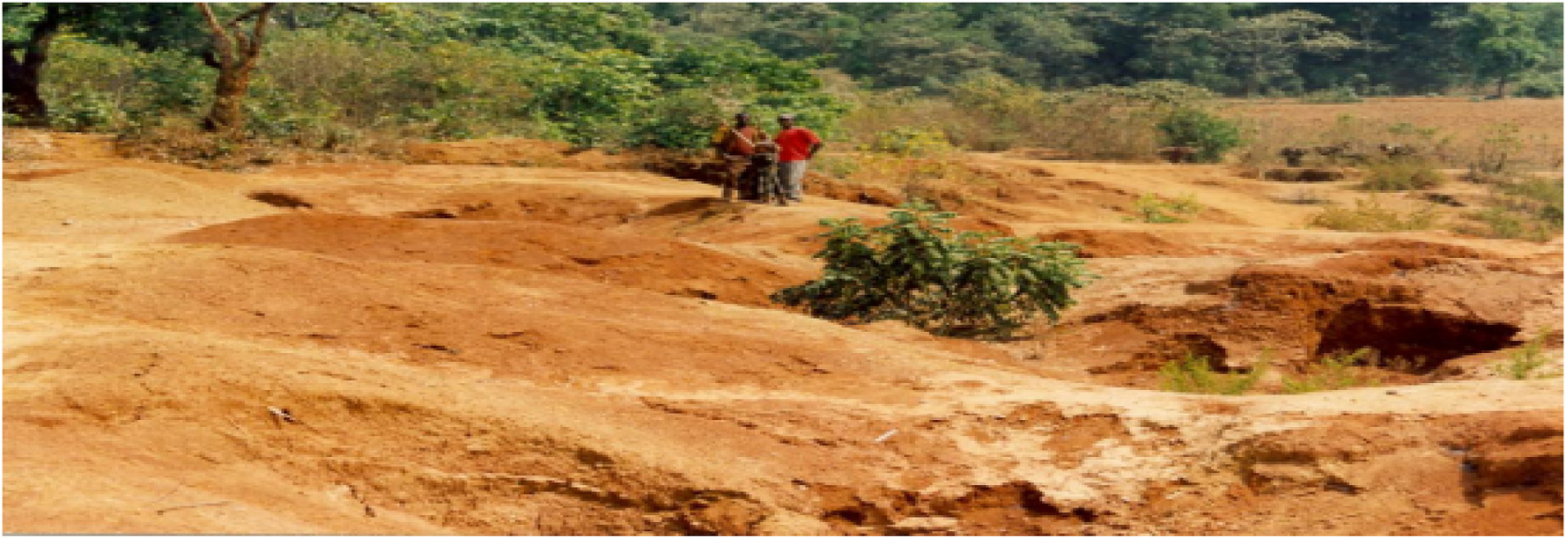
Shallow surface mining. Sources: Filed observation, 2021.

Also key informants replied that in addition to destroying the landscape, mining operations have a significant negative impact on the distraction of roads and other services in the study area. During the field survey, these have been perceived as small gold mining operations cause to lopsided destruction of the roads in the study areas (Figure 4). Most of the underground and surface mining operations, particularly in the small-scale local mining operations, look untidily erected, and the quarried materials that were transported to dangerous deep pits were reinforced feebly by wood and twigs (Jacob et al., 2020). Shallow mining operations for small scale gold often fail to support crop production due to loss of soil nutrients from topsoil (organic horizon) through erosion and finally leave the bare land (Figure 5). For instance, uncontrolled large-scale and small-scale mining without proper repossession also leads to further dilapidation of the landscape (Basuki, 2017).

The natural resources experts of the *Shekiso* district disclose that the environmental effects of the mining operation on local gold mining societies must be mitigated by destructive mining operations that worsen the environments where they operate. Also they reported that Adoption of agroforestry practices, reforestation, contour plowing, and increasing education about the effects of mining on the environment should be implemented to reduce environmental effects. Environmental policies should be formulated to support and motivate the local organizations, communities, and individuals who manage mining resources, and it is better to formulate legal instructions to rebuke those who abuse and exploit natural environments through mining operations. Furthermore, it should be necessary to avert, reduce, and modify the impacts of mining activity on environments such as water, landscape, natural vegetation, (Abdurashidovich, 2020).

According to the key informants, the main factors that determined the degradation of mining land in the study area were: lack of local community involvement in land management practices, lack of environmental rules and regulations, difficulties in recording small and large scale mining activity, lack of environmental education, etc. This finding is confirmed by Christianawati et al. (2020) whose study revealed that there were no nationally recognized standards for institutions for mining regulatory frameworks.

On the other hand, the shallow surface mining land was well recognized by extensive clearing of vegetation; consequently, severe demolition of the landscape and physical explosives to break down the rocks exposed water pollutants from dust and fumes (Julzarika et al., 2018). Shallow surface mining practices have a negative effect on changing the local scenery in the study area (Figure 5).

Moreover, in the process of gold extraction through the biological oxidation process, certain poisonous gases are emitted (Ismail et al., 2019). For example, sulphide, carbon, trioxides, and cyanide are highly toxic and harmful gases for human health are released into the air. This chemical events can sometimes be hazardous to workers and those in nearby communities (Campanale et al., 2020). Furthermore, toxic wastes produced by these processes are dumped into tailing dams, which are also point sources of land and water pollution in neighboring communities (Ohiozebau et al., 2017).

According to the survey results, the majority of respondents said that mining serves as a source of income (53%). It is followed by 30% of respondents reporting that mining is a main source of raw material for making ornaments and the remaining 17% of households said that mining is a source of employment for local communities in the study area (Table 5).

**Table 5:** Contribuation of Mining to local Households.

Response	Frequency	Percent
Sources of income	150	53
sources raw material for ornaments	83	30
Sources of employment	50	17

Source: household survey, 2021

### 3.4 Households' perception of gold mining

According to Funoh (2014), the mining industry requires technology, capital, and specialized skills for surplus mining operations. Due to mining technology, private or public mining companies may generate more revenue than traditional mining occupations (Haddaway et al., 2019; Hinton et al., 2003; Lahiri-Dutt et al., 2014; Werthmann, 2009). Artisanal and gold mining can help to alleviate poverty and create several possibilities (Baddianaah et al., 2021; Stephens and Ahern, 2001). However, it necessitates cash and specific abilities, which can be problematic for resource-constrained and inexperienced households without competent technical assistance.

According to the focus group discussion about 100%, stated that gold mining firm does not conduct needs assessments, including a variety of community residents, and environmental and social implications are not effectively assessed. As a result, the majority of households had a negative attitude toward such behaviors. About 14.28% of households have awareness to the effects of gold mining on enviroments, were as reminig 85% of respondants stated mining as a labor-intensive activity (Table 6). Houever, mining companies may not be able to particularly address the expectations of local communities if there are no defined guidelines on corporate social responsibility.

**Table 6:** Households' perception of gold mining.

	Statements	Disagree	Strongly agree	Neutral	Strongly disgree	Agree
1	The gold mining company conducts needs assessments of the community by involving a variety of community members				14 (100)%	
2	The company do not conducted study on environmental and social impacts				4 (28.5%)	10 (71.5%)
3	It increase the chance of land slide				4 (28.5%)	10 (71.5%)
4	The gold mining produce some dangerous substance to enviroments		2 (14.28%)	6 (42.8%)	6 (42.8%)	
5	The gold mining company does organize some workshop for awareness creation about effects of gold mining on enviroments		8 (57%)		2 (14.28%)	4 (28.5%)
6	It incerase enviromental pollution		3 (21.42%)			11 (78.5%)
7	It cause to land degration					14 (100%)
8	It is capital intesive activity		1 (7%)	2 (14.2%)	11 (78.5%)	
9	It is labour intesive activity			12 (86%)	2 (14.2%)	
10	It incrase soil erostion		1 (7%)	1 (7%)	12 (85.7%)	

Source: household survey, 2021

As a result, traditional mining produced less revenue and required more labor, leading to a poor perception of mining. According to the survey results, 43% of total households had a positive attitude toward mining activities in the study area, while the remaining 57% had a negative attitude (Table 7). While mining is commonly thought of as a labor-intensive activity requiring significantly more money than farming, the questions looked at both the negative and positive aspects of the relationship, as well as opinions on why mining failed to create revenue and the people involved returned to farming. While our approach may be comparable to that of who sought scenarios where artisanal mining revenues endorsed agriculture in Sierra Leone, there is one substantial difference: the situations we encountered in Zimbabwe tended to show the reverse movement of investment, with gold mining widely seen as the increasingly dominant source of income.

**Table 7:** Households’ perception to gold mining.

Perception to gold mining	Frequency	Percent
Positives	123	43
Negative	160	57
Total	283	100

Sources: Household survey, 2021

According to the survey, gold mining was employed in all three sub-locations of the research region (Lilian et al., 2016). This is clear based on current observations in the research region as the most typical feature of the houses. For the great majority of artisan miners, mining is their principal source of income (legal and illicit). It is the main source of extra income in the study area (Hong and Sullivan, 2013). According to key informants and focus group members, artisan mining provides an average of 74% of miners' income (N et al., 2011). In a situation where various actors had varying interpretations of the region's mining past, the focus group discussion designed for this study focused specifically on how relocated farmers saw their engagement and investments in mining in the years that followed (Mkodzongi et al., 2019).

## 4 Conclusion and recommendation

This study assesses the effects of gold mining on the environment: Shekiso District, Guji Zone, Ethiopia. To arrive at the stated objectives, the researcher applied cross-sectional survey research design and used mixed approaches. A probable and non-probable sampling technique was needed to select the sample households. To collect the necessary information, the researcher used both primary and secondary data sources. Key informant interviews, questioners, observation, and focus group discussions were used to collect data. The 283 total representative sample households was included for sample. Both quantitative and qualitative data were gathered from households. The survey result shows that mining possesses a negative environmental impact on households, and the perception of gold mining is widely believed to be negative due to several reasons. Moreover, the study area faces several environmental challenges are caused by unsustainable mining operations, such as a shortage of water (8.8%), dehydration of the brook (10.6%), soil erosion (20.8%), and destruction of the ecosystem (7.0%), etc. This implies a lack of continuous follow-up maintenance and bendable planning systems. The key informant was also remarked upon as households are mindful of the effects of gold mining activities on their environmental quality. In line with the seriousness of the environmental problems resulting from gold mining, local communities were eager to embrace and contribute to restoration tasks. It indicates that when a “bottom-up” approach is used, communities are incorporated into essential players in the re-establishment of degraded environments, and success rates may be higher than they would be otherwise. To a large extent, the accountability and authority of the gold-extracting industry for the impact of environmental degradation in the study area have been very poor. As a result, the study's survey results are used to generate valuable information for policy formulation and to improve sustainable mining by reducing the risk of mining in the environment.

The findings recommended that the offices of environment and natural resources management closely monitor gold mining environments to ensure they minimize the negative impact of mining on the environment. Priority should be given to encouraging open investment to further research designed to improve our understanding of the effects of mineral extraction on land degradation and ways to boost environmental sustainability in the study area.

Lastly, advancing the 2030s sustainable development goals deeper transitions to more resource-efficient, resilient forms of growth that bring social, economic and environmental benefits in longer term. This requires a focus on, benefit from a healthy environment, regarding to the effect of gold mining environments. The government has to evaluate the existing legislatives and close the gap clear commands and guding principles on gold mining. Human Implications the effect caused by toxic chemical pollution on the nearby community should be compensated. Moreover, government has to make local community employment creation activity as a mandatory act for licensing and relicensing. To redress the cultural disorientation created due to the activity of gold mining, were the cultural Development and Truism Office should have to work with community elders and Abba Gedas. The environmental rehabilitation and community development programs should be, enhanced and enforced by local government and others concerning bodies in study area. Finally, clearly agreement should be made among mining companies at the federal level to the regional and local govern mining resources as environmentally sustainable.

## Declarations

### Author contribution statement

Birhanu Bekele Mencho, M.d: Conceived and designed the experiments; Performed the experiments; Analyzed and interpreted the data; Contributed reagents, materials, analysis tools or data; Wrote the paper.

### Funding statement

This research did not receive any specific grant from funding agencies in the public, commercial, or not-for-profit sectors.

### Data availability statement

Data included in article/supp. material/referenced in article.

### Declaration of interest's statement

The authors declare no conflict of interest.

### Additional information

No additional information is available for this paper.
